# Lepromatous Leprosy with Crusted Scabies

**DOI:** 10.4269/ajtmh.20-0763

**Published:** 2020-12

**Authors:** Raihan Ashraf, Tarun Narang, Muthu Sendhil Kumaran

**Affiliations:** Department of Dermatology, Venereology, and Leprology, Postgraduate Institute of Medical Education and Research, Chandigarh, India

**Figure 1.**
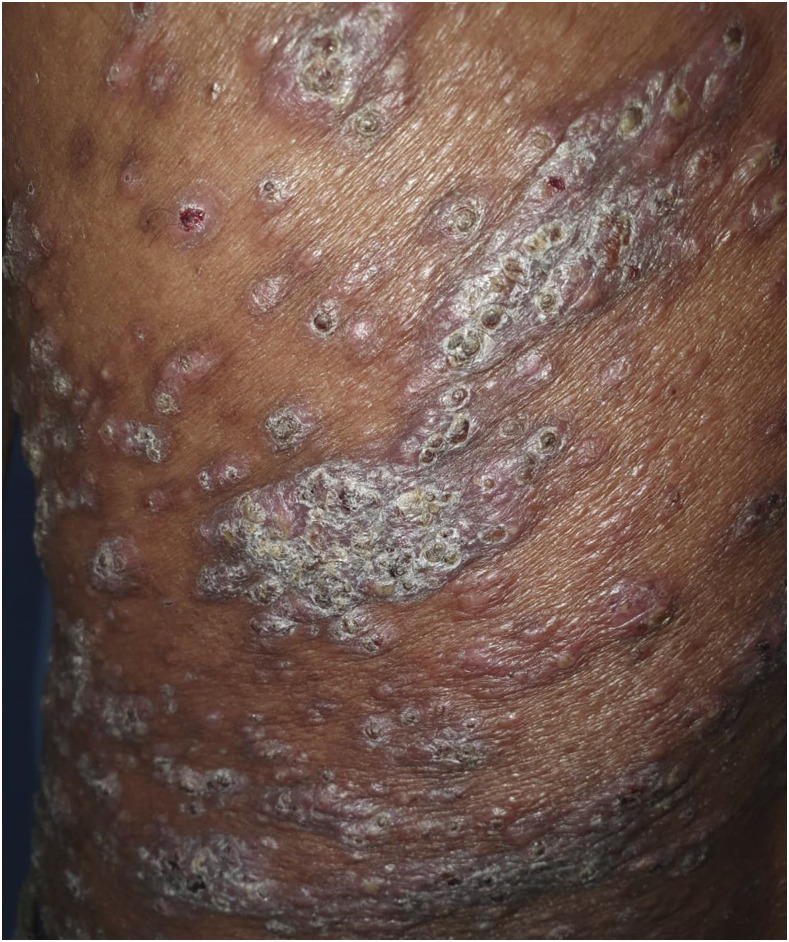
Grouped crusted papules and plaques on the trunk of the patient on a background of infiltrated skin. This figure appears in color at www.ajtmh.org.

**Figure 2.**
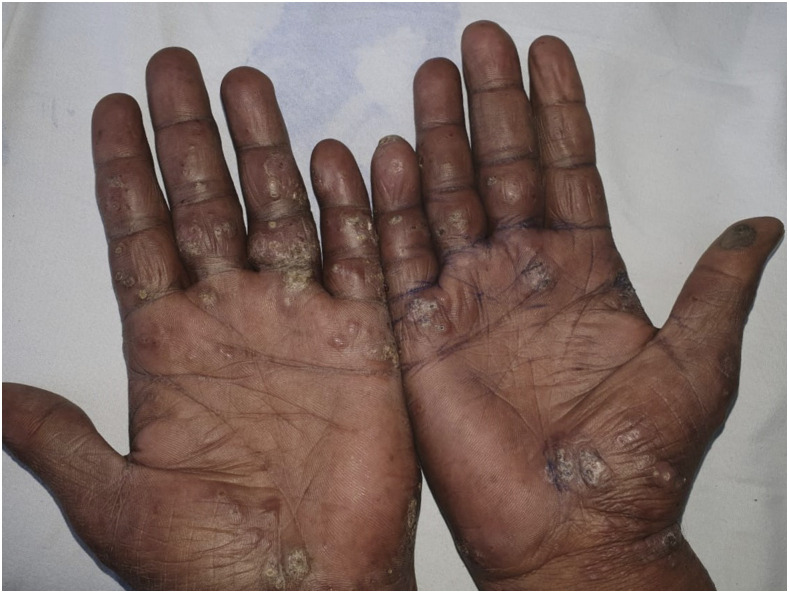
Erythematous papules and nodules with overlying small crusts on palms and fingers of the patient. This figure appears in color at www.ajtmh.org.

**Figure 3.**
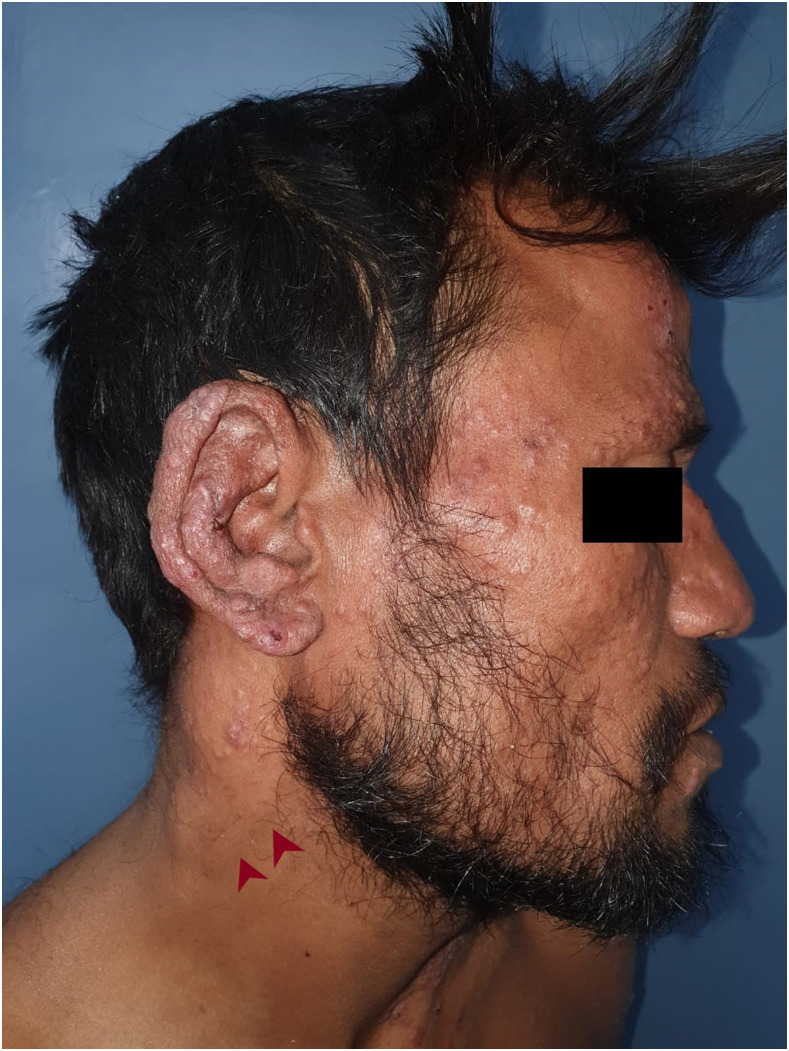
Discrete erythematous papules may be seen on the face and neck, whereas they are coalescent and crusted on the ear helix. There is infiltration of the skin on the face and ears, with madarosis and depressed nasal bridge. Thickened greater auricular nerve is visible on the neck (red arrowheads). This figure appears in color at www.ajtmh.org.

**Figure 4.**
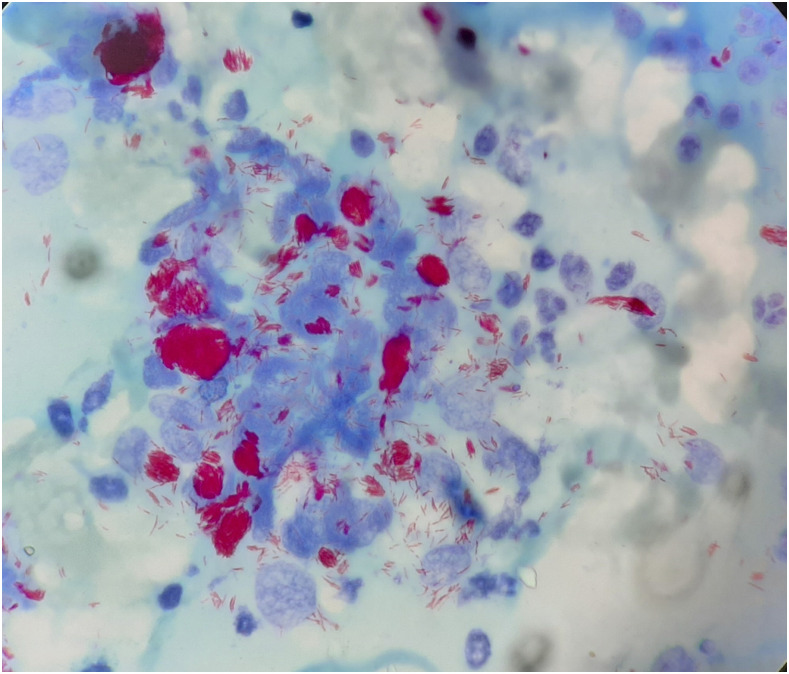
Ziehl–Neelsen staining of slit-skin smear revealing the presence of acid-fact bacilli in groups and as globi (bacteriological index -6+; ×100). This figure appears in color at www.ajtmh.org.

**Figure 5.**
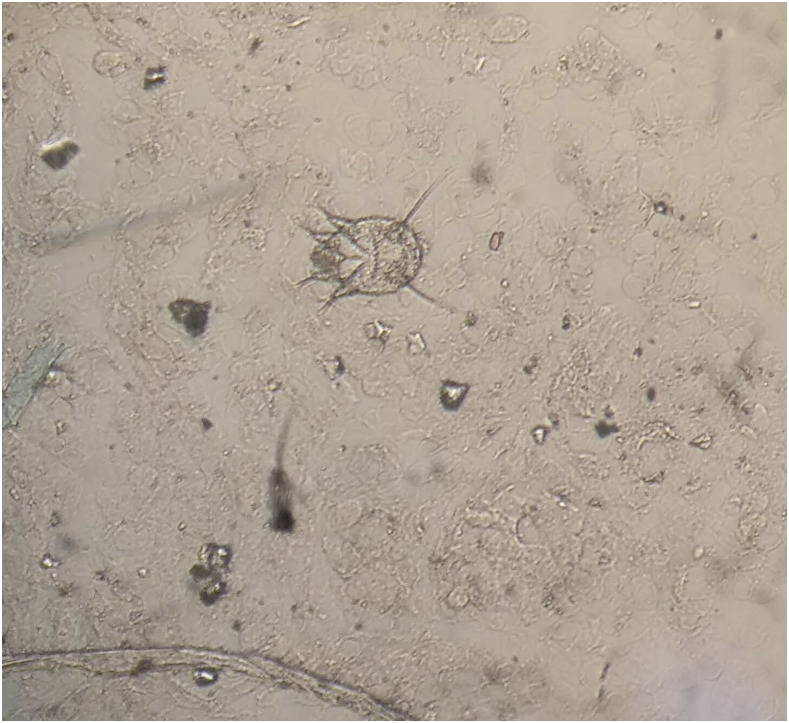
Scabies mite on KOH mount (×40). This figure appears in color at www.ajtmh.org.

A 36-year-old vegetable vendor presented with asymptomatic crusted lesions on the skin of 2-month duration. On examination, there were grouped crusted papules and plaques all over the body (Figure 1), including palms (Figure 2), soles, ear helix (Figure 3), and genitals, on the background of infiltrated skin. Nervous system examination revealed thickened peripheral nerves and a glove-and-stocking pattern of sensory loss without any motor weakness. Slit-skin smear from the papules on the trunk showed acid-fast bacilli (bacteriological index -6+; [Figure 4]), and potassium hydroxide mount of scrapings from hands and the trunk revealed scabies mite (Figure 5). The test for HIV was negative. A diagnosis of lepromatous leprosy with crusted scabies (CS) was rendered, and he was started on an anti-scabetic regimen for CS as per CDC guidelines^1^ (daily topical 5% permethrin application for 7 days followed by twice weekly for 2 weeks, with oral ivermectin 12 mg on days 1, 2, 8, 9, and 15) along with the WHO multidrug therapy–multibacillary regimen for leprosy (monthly supervised doses of rifampicin 600 mg, dapsone 100 mg, and clofazimine 300 mg, followed by daily dapsone 100 mg and clofazimine 50 mg, for 12 months) with improvement in symptoms. All close contacts were screened for leprosy and treated for scabies as well.

Crusted scabies is a highly contagious variant of scabies wherein the host immune response fails to control the proliferation of the mites in the skin, resulting in hyper-infestation and an inflammatory reaction. It is seen mostly in immunocompromised elderly or physically incapacitated individuals. Leprosy has been among the diseases that predispose to CS, hypothesized to be due to a predominant T-helper-type of immune response, especially in lepromatous leprosy.^2^ In addition, overcrowding and poor socioeconomic conditions are predisposing factors for both diseases. In a study of scabies in elderly patients with a history of leprosy, 66% belong to the lepromatous spectrum.^3^ Another study of 78 patients with CS reported 17% of patients to have had leprosy.^2^

Both leprosy and scabies are neglected tropical diseases. Leprosy in addition is associated with significant stigma and discrimination. These patients are often poor and neglected by their own families and the society at large, predisposing them to other infections and infestations such as scabies, adding to their overall morbidity.
